# The cytoplasmic 60 kDa progesterone receptor isoform predominates in the human amniochorion and placenta at term

**DOI:** 10.1186/1477-7827-7-22

**Published:** 2009-03-13

**Authors:** Anthony H Taylor, Penny C McParland, David J Taylor, Stephen C Bell

**Affiliations:** 1 Preterm Birth Research Group, Reproductive Sciences, Department of Cancer Studies and Molecular Medicine, University of Leicester, Leicester, Leicestershire, LE2 7LX, UK

## Abstract

**Background:**

The mechanism that initiates human parturition has been proposed to be 'functional progesterone withdrawal' whereby the 116 kDa B-isoform of the progesterone receptor (PR-B) switches in favour of the 94 kDa A-isoform (PR-A) in reproductive tissues. Recently, other PR isoforms, PR-S, PR-C and PR-M generated from the same gene have been identified and partially characterised.

**Methods and Results:**

Using immunohistochemical, western blotting and RT-PCR techniques, evidence is provided that indicates the major PR isoform present in human term fetal membranes (amnion and chorion) and syncytiotrophoblast of the placenta is neither of the classical nuclear PR-B or PR-A isoforms but is the N-terminally truncated 60 kDa PR-C isoform. Evidence is also provided that this 60 kDa isoform resides in the cytoplasm of the expressing cell types. Data are also presented to show that PR-B, PR-A and PR-S isoforms are essentially absent from the amnion and chorion, whereas PR isoforms A, B, C and S are all present in the decidua, with PR-A being the major isoform. The syncytiotrophoblast of the placenta contains the cytoplasmic 60 kDa isoform, but not isoforms PR-A, PR-B or PR-S.

**Conclusion:**

The major PR isoform in the amnion, chorion and placenta is a 60 kDa protein that could be PR-C, suggesting that the cytoplasmic isoform has a specific role in extra-embryonic tissues and may be involved in the regulation of human parturition.

## Background

Progesterone receptors (PRs) are members of a superfamily of ligand-activated nuclear transcription factors comprised of specific domains involved in DNA binding, hormone binding, and transactivation [1]. Progesterone activation of PR in target tissues is mediated via dimerisation and phosphorylation of the receptor, resulting in binding to *cis*-acting progesterone response elements on DNA and the modulation of the promoters of target genes [1,2]. The human PR-A isoform differs from the PR-B isoform in lacking the first 164 amino acids contained in PR-B [3]. Both are translated from distinct mRNA transcripts generated from a single gene under the control of separate oestrogen sensitive promoters [4]. Previous work has identified three additional AUG codons that act as translation sites with a possible methionine site at amino acid 595 that is predicted to generate a protein of approximately 60 kDa [5,6]. More recently, two additional translational start sites at amino acids 289 and 301 have been identified that also produce proteins of approximately 60 kDa [7].

Although much work has been performed on PR-B and PR-A, little work has been undertaken on the other seven transcripts generated from the PR gene [5], despite there being evidence that some of these are translated into functional 38 kDa, 60 kDa, 71 kDa or 78 kDa proteins in malignant progesterone target tissues [8,9] and that these are co-ordinately up-regulated by oestrogens and down-regulated by progestins [10,11]. Evidence also exists for other PR isoforms such as PR-C, PR-S and PR-T, which could be genomic mediators of progestin action [12,13] and for three membrane progestin receptors that are classical G-coupled protein receptor-transduction molecules first identified in the teleost oocyte called mPRα, mPRβ and mPRγ [14,15].

Progesterone receptors have been proposed to play a key role in the control of human labour and parturition whereby the levels of the PR-B isoform, which is often considered to be the dominant isoform, fall prior to and during labour leaving the PR-A isoform as the predominant form leading to a 'functional progesterone receptor withdrawal' [16]. Evidence to support this occurring in the uterine myometrium exists [17], although more recent evidence suggests that the PR-C isoform is also expressed and may have a functional role [18,19]. In other human reproductive tissues, such as the decidua, ovary and the oviduct [20,21], PR-A appears to be the predominant progestin regulator with PR-B maintaining a supporting role suggesting that progestin signalling in the human uterus at the end of parturition is far more complex than a PR-B to PR-A isoform switching mechanism [17]. Despite there being a paucity of data to support 'functional progesterone receptor withdrawal' in tissues at the fetal-maternal interface, i.e. in the fetal membranes, decidua and placenta, many still consider that only the PR-B and PR-A isoforms are present [22,23].

Recent data have suggested that at least five nuclear PR-isoforms are present in the human decidua and that all five isoforms are decreased after labour [24,25]. However, although western blotting techniques also indicated the presence of several PR isoforms in amniotic nuclear extracts, immunohistochemical methods failed to detect any PR isoforms in the amnion and chorion [25].

In the present study examining the pattern of expression of PR isoforms in human fetal membranes (amnion and chorion), decidua and placenta at term, we demonstrate that the major isoform present in the fetal membranes and placenta is a cytoplasmic 60 kDa isoform, an isoform of molecular mass identical to that of PR-C, that PR-B or PR-A are not expressed in the amnion or chorion, and that the 94 kDa PR-A protein is the dominant PR isoform in the decidua.

## Methods

### Patient samples

Local Research Ethics Committee approval for the study was obtained and all patients signed informed consent for their tissues to be used. Fetal membranes and placenta (n = 6) were collected from term patients undergoing elective Caesarean section prior to labour. All tissues were divided into 3 parts; one fixed in formalin and embedded in paraffin for histological examination, the other two snap-frozen in liquid nitrogen and stored at -80°C for later RNA and protein extraction. Enriched amnion was obtained by careful peeling of the amnion away from the chorion and decidua. Enriched decidua was obtained using the edge of a microscope slide to carefully remove a thin layer from the inverted fetal membranes. Enriched chorion was obtained by using the edge of the microscope slide to scrape away the remaining decidua. A 2 cm^3 ^block of placenta from the mid-part of a cotyledon was taken and washed briefly with sterile PBS before division into 3 parts.

### Immunohistochemistry

Five μm sections of tissue dried for 48 hr prior to de-waxing and re-hydration through graded alcohol to distilled water were subjected to boiling 10 mM citric acid for 12 min followed by cooling for exactly 20 min before transfer into cold H_2_O to retrieve antigenic sites. Endogenous peroxidase activity and specific binding was blocked with 6% peroxide for 15 min, followed by incubation with non-immune rabbit serum (10% in phosphate buffered saline (PBS)) for 30 min, respectively. Endogenous avidin and biotin sites were blocked with Avidin-Binding blocking solutions (Vector Laboratories, Peterborough, UK) according to the manufacturer's instructions. Antibodies that detect all known isoforms of PR, clone 1A6 and C-20, were purchased from Novacastra Laboratories, Newcastle-upon-Tyne, UK and Santa Cruz Biotechnologies, Santa Clara, CA, USA, respectively. The antibodies that detect only PR-B (San27) and antibodies that detect only PR-A (clone 16) in immunohistochemistry were purchased from Novacastra. Monoclonal antibodies diluted in 10% non-immune rabbit serum, at 1:50 (1A6), 1:150 (San27), 1:200 (clone 16) dilutions, and the polyclonal antibody C-20 diluted to 1:40 in 10% swine serum, were applied to sections and incubated overnight at 4°C. In some studies, C-20 antibodies were pre-incubated (3 hr at room temperature) with a 7-fold excess of immunising peptide before use. After thorough washing in PBS, biotinylated rabbit anti-mouse or swine anti-rabbit secondary antibodies (1:400) were applied for 1 hr 30 min, the sections washed and avidin-biotin complexes applied for 30 min. Colour was developed over antigenic sites using 3, 3'-diaminobenzidine for 5 min. After copious washes in deionised water, sections were lightly counterstained with haematoxylin, dehydrated through graded alcohols, cleared in xylene and permanently mounted in XAM (BDH, Poole, UK). Photomicrographs were obtained at the indicated magnifications on a Zeiss Axioplan compound microscope (Zeiss, Welwyn Garden City, UK) fitted with a Sony DN-100 digital camera (Nikon, Kingston-upon-Thames, UK). Images were digitally enhanced using proprietary software.

### RNA preparation and RT-PCR

Total cellular RNA was obtained from 100 mg of tissue using Trizol reagent (Invitrogen, Paisley, UK) according the manufacturer's instructions, and genomic DNA contamination removed by treating the samples with RNase-free DNase 1 (Promega, Southampton, UK) for 1 hour at 37°C, followed by phenol-chloroform extraction and isopropanol precipitation. After verification of RNA quality by UV-spectrophotometer analysis and agarose gel electrophoresis, one μg of RNA was reverse transcribed using avian myeloblastosis virus reverse transcriptase (AMV-RT) for one hr at 42°C. A minus AMV-RT control was obtained by substituting diethylpyrocarbonate (DEPC)-treated water for the AMV-RT enzyme.

PCR was performed using PR primer combinations that identified all PR isoforms, the PR-B isoform, the PR-A and PR-B isoforms or the PR-S isoform [26]; (Table 1).

**Table 1:** Designation and sequences of primers used in RT-PCR.

Primer	Sequence	Combinations	Size (bp)	Detects
pB5'	CCTGAAGTTTCGGCCATACCT	p15 & p35	284	B, A, C, M & S
pB3'	AGCAGTCCGTGTCCTTTCT	p33 & p36	396	B & A
pS	GAATTCAGGAGAGTGGGTGCTC	pS & p33	186	S
p15	AGGAGTTTGTCAAGCTTCAA	pB5' & pB3'	197	B
p33	GAATTCATTTGGAACGCCCACTGG			
p35	CTGCAGGGACTGGATAAATGTATTC			
p36	CTGCAGGTCTACCCGCCCTATC			
gapdf	AGAACATCATCCCTGCCTC	gapdf & gapdr	347	GAPDH
gapdr	GCCAAATTCGTTGTCATACC			

Primer combinations, expected amplicon size, reaction conditions and isoforms detected are shown in Fig 2 and are described in references 26 and 33. GAPDH, Glyceraldehyde-3-phosphate dehydrogenase.

### Western blotting

Protein extracts were obtained by homogenising samples in ice-cold lysis buffer (1% Igepal, 0.5% sodium deoxycholate, 0.1% sodium dodecyl sulphate (SDS), 2 mM EDTA, 50 mM sodium fluoride, 1 mM sodium metavanadate, 0.2 mg/ml aprotinin all from Sigma-Aldrich, Poole UK, in PBS (pH 7.0) but without phenyl methyl sulphonyl fluroride) [27]. After incubation on ice for 30 min, insoluble material was removed by centrifugation at 14,000 g for 20 min at 4°C and supernatants stored at -20°C. One hundred μg of protein per lane was resolved by 7.5% SDS-polyacrylamide gel electrophoresis, transferred to nitrocellulose membranes (Amersham-Pharmacia Biotech, Chalfont St Giles, UK) and probed with anti-PR antibodies 1:50 (clone 1A6) or 1:400 (C-20) in 3% Blotto. Peroxidase-conjugated secondary antibody (Amersham) was used at a 1:2000 dilution and the reaction visualised using ECL detection kits. Negative controls were performed with blots not exposed to primary antibody and by incubation of antibodies with a 7-fold excess of immunising peptide or extracts produced from choriocarcinoma (BeWo) cells transiently transfected with the human PR-C [13] expression plasmid.

## Results

**Figure 1:**
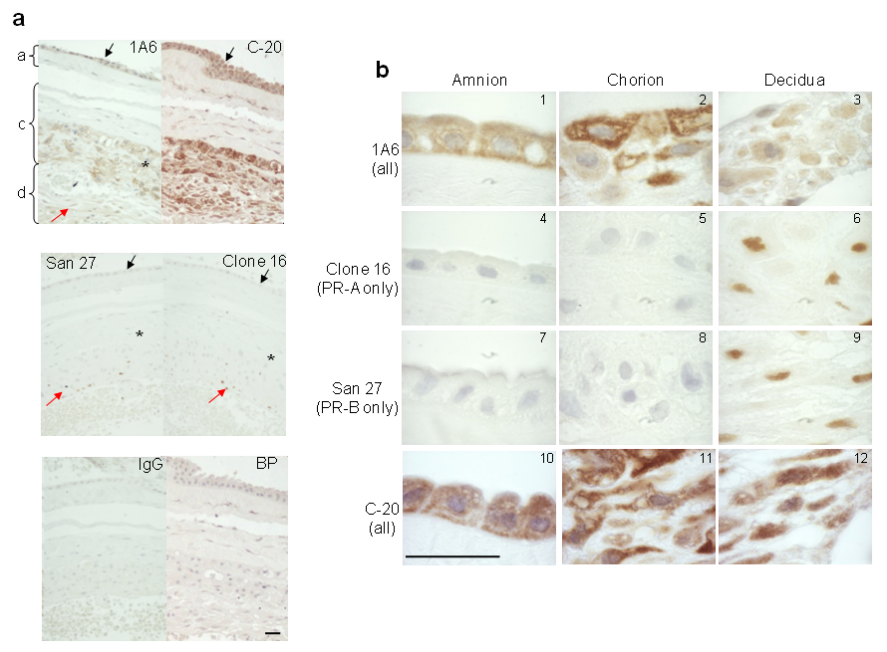
**Identification of PR-positive cell types at the fetal-maternal interface**. (**Panel a**) Low power image of fetal membranes showing the immunohistochemical staining patterns obtained with antibodies that detect all isoforms of PR (clone 1A6) and (C-20), antibodies that detects only PR-B (San27) and antibodies that detect only PR-A (clone 16). An equivalent amount of mouse immunoglobulins (IgG) or antibody pre-adsorbed with immunising peptide (BP), against which C-20 antibodies were generated, indicates specificity. Immunoreactivity is indicated with the black arrows in the amniotic (a), the star in the chorionic (c) and with red arrows in the decidual (d) layers of the fetal membranes. (**Panel b**) The presence of cytoplasmic PR and an absence of PR-B and PR-A isoforms are demonstrated in term amnion epithelial cells (**1, 4**, **7**, and **10**), and chorionic cytotrophoblasts (**2**, **5**, **8**, and **11**). Both cytoplasmic and strong nuclear staining was observed in the decidua (**3**, **6**, **9 **and **12**) indicating the presence of cytoplasmic and nuclear PR isoforms. Bar = 50 μm; Data are representative of six independent samples.

**Figure 2:**
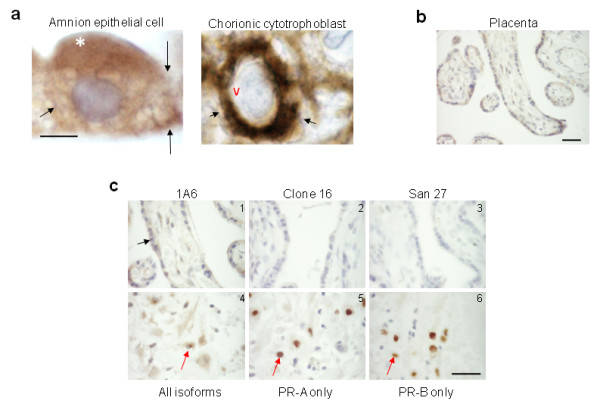
**Cellular localisation of PR isoforms**. (**Panel a**) High power images of PR immunostaining with clone 1A6 indicating immunoreactivity in the cytoplasm of the amnion epithelial cell and the chorionic cytotrophoblast. Note the absence of staining in the blue nucleus of either cell type. The plasma membrane of the cell is shown by the arrows, the apical surface by the white asterix and a void between the nucleus and the cytoplasm by the red V. (**Panel b**) A low power image of PR immunostaining of the term placenta with clone 1A6 antibodies indicating immunoreactivity in the syncytiotrophoblast layer (arrow), but absent elsewhere. (**Panel c**) High power images of PR immunostaining of term placenta indicating the presence of cytoplasmic PR in the syncytiotrophoblast but not nuclear PR-B and PR-A isoforms (**panel c; 1, 2, **and **3**); whereas basal plate decidual cells contained both cytoplasmic and nuclear PR isoforms (**4, 5, **and **6 arrowed**). These data suggest the cytoplasmic PR staining in amnion epithelial cells, cytotrophoblasts, syncytiotrophoblasts and decidual cells, is not PR-B or PR-A, but PR-S, PR-M or PR-C. Bar = 50 μm, except panel A where Bar = 5 μm. Data are representative of six independent samples.

To investigate the PR isoform repertoire of the progesterone-dependent human fetal-maternal interface, immunohistochemical analysis using the commercial PR antibody clones 1A6 and C-20 generated towards the ligand- and DNA-binding domains, respectively, of the full length PR-B isoform were used (Figs 1 and 2). The data revealed the presence of cytoplasmic staining in the amnion epithelial cell, the chorionic trophoblast and the maternal decidual cell. Nuclear staining with these antibodies was only found in decidual cells and positive control tissues such as breast cancer, endometrium and myometrium (positive control data not shown). No nuclear staining was detected in the amnion epithelium and chorionic cytotrophoblast (Figs 1 and 2). The connective tissue cells of the reticular and chorionic layers of the fetal membrane were devoid of any immunoreactivity (Fig 1a). In the placenta, the syncytiotrophoblast also demonstrated cytoplasmic staining with this antibody and no nuclear staining (Fig 2a). However, cytoplasmic and nuclear immunoreactivity was observed in decidual cells attached to the basal plate (Fig 2a).

To confirm the identity of the cytoplasmic isoform found in these cell types, immunostaining with PR-B- and PR-A-specific antibodies revealed nuclear staining in the decidual cell, but no other staining elsewhere in the fetal membrane (Figs 1 and 2). Similarly, nuclear staining was confined only to the decidual cells of the basal plate attached to the placenta (Fig 2c). Nuclear staining in classical progesterone target tissues (breast cancer, endometrial, and myometrial cells) confirmed the PR-B and PR-A specificity of the antibodies (data not shown). The conclusion from these studies was that the cytoplasmic isoform found within fetal membranes, decidua and the syncytiotrophoblast of the placenta was neither the PR-B nor the PR-A isoform and could represent one of the other PR isoforms. These data were inconsistent with previous observations that the amnion epithelial cell contained both PR-B and PR-A isoforms and that during labour there is a loss of the PR-B isoform in favour of the PR-A isoform [23], but is consistent with the immunohistochemical studies of Goldman et al. [25].

**Figure 3:**
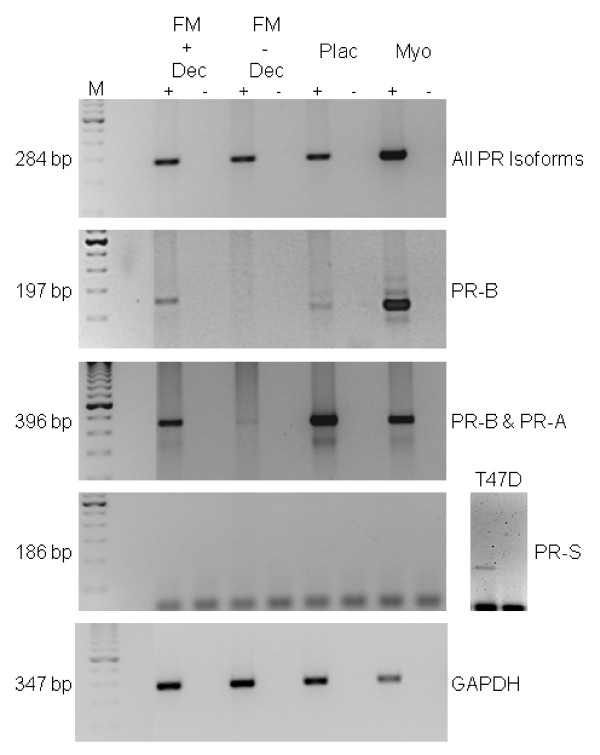
**Evidence that the PR isoform in human fetal membranes and placenta at term is either PR-C or PR-M**. RT-PCR products generated from fetal membrane samples that had decidua attached (**FM + dec**) or fetal membranes that had the decidua scraped away (**FM – dec**) or term placenta (**Plac**). One μg of DNase-treated total RNA was reverse transcribed in the presence (+), or absence (-), of AMV-RT. Both samples were then amplified in a PCR with isoform-specific primers (see Table 1) that detected all known PR isoforms; the PR-B isoform alone; a combination of PR-A and PR-B; or the PR-S isoform alone. GAPDH was used a control for efficiency of mRNA manipulation and PCR amplification. Myometrium (**myo**) was used as a positive control for PR with T47D cell extract as a positive control for PR-S (**separate column**), and in all cases a minus RT sample was incorporated to rule out the presence of contaminating genomic DNA. Results are representative of four independent assessments. Data show the presence of PR in all 4 tissue samples and T47D cells.

**Figure 4:**
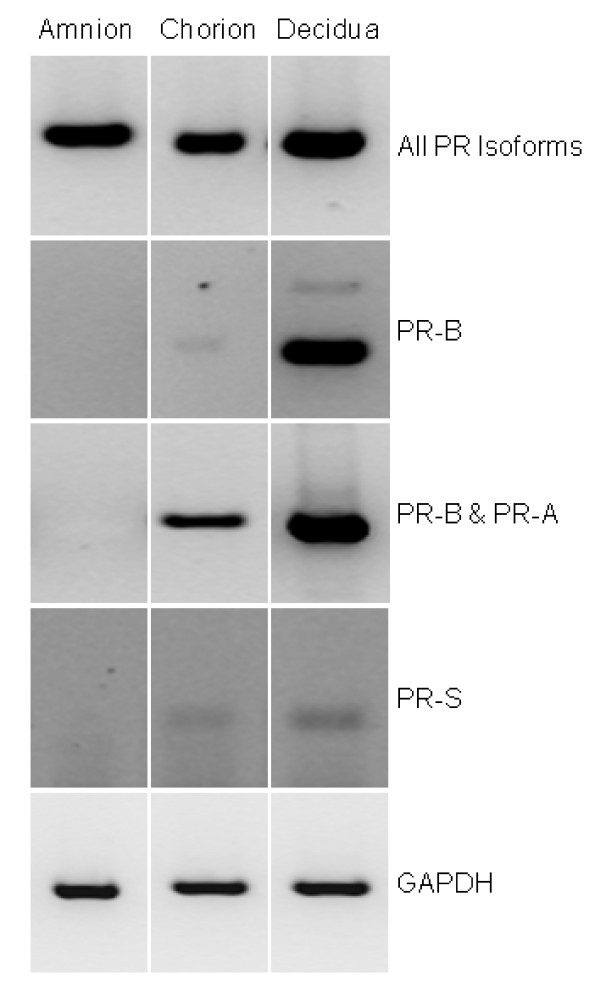
**RT-PCR gels showing the PR transcripts found in extracts of enriched amnion (amnion), chorion (chorion) and decidua (decidua)**. Data were obtained from the +RT sample only and indicate that the amnion contains a PR transcript that is not PR-A or PR-B. Additionally, enriched decidua contains the PR-S isoform whereas the chorion (which is contaminated with decidua) also contains PR-S, PR-B and PR-A. The amnion does not contain these transcripts, but does contain a PR isoform. The bands visible at the bottom of the 186 bp PR-S isoform gel are unused primers.

To find the identity of the cytoplasmic progesterone receptor isoform, RNA extracts prepared from term fetal membrane and placenta samples were subjected to RT-PCR with several isoform-specific primer sets (Table 1) and revealed the presence of a PR transcript in all samples by RT-PCR (Figs 3 and 4; top panels). Analysis with PR-B-, PR-A and/or PR-B- and PR-S-specific primer sets showed that fetal membranes with decidua attached consisted mainly of PR-A transcripts with some transcripts for PR-B, but no PR-S transcripts (Figs 3 and 4). Fetal membranes enriched for amniochorion revealed the presence of a transcript for PR that was not PR-B, not PR-A or PR-S leading to conclusion that the cytoplasmic PR isoform present was either PR-C or PR-M previously observed in human breast cancer cells and isolated from human aortic endothelial cells, respectively [9,13,28,29]. The placenta similarly contained a transcript for PR that was mainly PR-A, although transcripts for PR-B were also detected (Figs 3 and 4). Transcripts for PR-S were undetectable, except in the T47D breast cancer cell control. Samples enriched for decidua revealed the presence of all PR isoforms including PR-S (Fig 4), whereas the amnion lacked transcripts for PR-A, PR-B and PR-S and the chorion (which is often contaminated with decidua) produced a major band for PR-A, although small amounts of transcripts for PR-B and PR-S were also detected (Fig 4).

**Figure 5:**
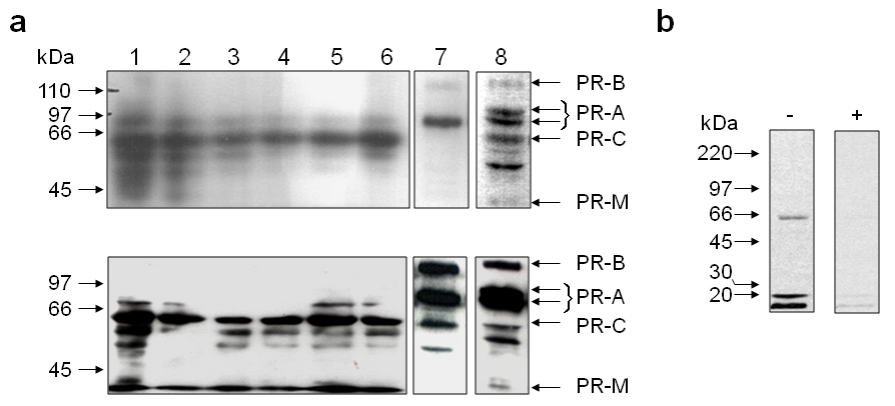
**Evidence that the major PR isoform in human fetal membranes at term is the 60 kDa isoform**. (**Panel a**) Protein extracts from fetal membrane samples that had decidua attached (**lane 1**) and fetal membranes that had the decidua scraped away (**lane 2**) were compared with extracts from term amnion (**lane 3**), chorion (**lane 4**) and decidua (**lane 5**) that were obtained from crude tissue separation methods and from term placenta (**lane 6**), myometrium (**lane 7**) and breast cancer (T47D cell) extracts (**lane 8**). The antibody clones used were 1A6 (upper series) and C-20 (lower series) that detect all PR isoforms (see lane 8). (**Panel b**) Confirmation that the antibodies detect the 60 kDa PR-C isoform was obtained by immunoblotting an extract obtained from human choriocarcinoma (BeWo) cells transiently transfected with a human PR-C expression plasmid and probed with C-20 antibodies (-) or C-20 antibodies pre-adsorped with immunising peptide (+).

When protein extracts from partially purified fetal membranes, placenta, trophoblasts and decidua were compared with those from T47D breast cancer cells, using western blotting techniques with the PR antibodies that detected the cytoplasmic isoform in the immunohistochemistry studies (Figs 1 and 2; Table 2), several PR immunoreactive proteins were observed in T47D breast cancer cell extracts in line with previous studies [6,11,25,27,30], whereas the tissues at the fetal maternal interface revealed a major PR isoform with a relative molecular mass of ~60 kDa (Fig 5). Other progesterone target tissues, such as endometrium (data not shown) and myometrium possessed the 116 kDa PR-B, 94 kDa PR-A and 60 kDa isoforms but only with the C-20 antibody, although weak bands were present with the 1A6 monoclonal antibody (Fig 5).

**Table 2:** Summary of PR isoform expression patterns

Tissue	PR-B^1,2,3^	PR-A^1,3^	60 kDa^1^,*	PR-S^2^	PR-M^3^
Amnion	-	-	+++	-	-
Chorionic cytotrophoblast	-	-	+++	-	N/D
Placental cytotrophoblast	-	-	-	-	N/D
Placental syncytiotrophoblast	-	-	+	-	N/D
Maternal decidua	++	+++	++	+	-

Presence determined by ^1^Immunohistochemistry, ^2^RT-PCR, or ^3^Western blot.

* The 60 kDa isoform is classified as cytoplasmic staining in immunohistochemistry; + weak presence, ++ moderate presence, or +++strong presence, N/D not determined.

The data indicate the presence of a major PR isoform of ~60 kDa in all the fetal membrane samples and that tissues 'contaminated' with decidua were devoid of significant levels of the PR-B but contained small amounts of PR-A isoforms that are easily detected in myometrium and T47D cell controls. Placenta and term decidua also indicated the presence of a ~60 kDa protein as the major PR isoform although other PR-isoforms were observed. Using the 1A6 antibody, the 38 kDa PR-M isoform was only observed in the tissues contaminated with deciduas and T47D extracts, whereas it was visualised in all samples with the C-20 antibodies.

## Discussion

The role of progesterone receptors in human gestation and parturition is to mediate the actions of progesterone so as to maintain pregnancy. In most mammals, the stability of the relationship in the fetal-maternal interface is disturbed by the fall of progesterone production by the placenta, with the concomitant softening of the cervix, rupture of the fetal membranes and initiation of highly synchronised high-pressure contractions of the myometrium that characterises labour [31]. In the human, similar events occur in the same coordinated manner except there is no decrease of systemic progesterone concentration [31]. A hypothesis presented [32], that a 'switch' in PR isoforms from the more transcriptionally dominant PR-B to the less active PR-A isoform must therefore occur in these tissues, has been convincingly supported by evidence in the myometrium [32,33], and in the cervix [34,35] and less convincingly in the amnion and maternal decidua [25].

In the present study we have shown that human fetal membranes (amnion epithelial cell and chorionic cytotrophoblast) contain mainly high levels of a 60 kDa PR isoform (Figs 1 and 2; Table 2) that could be PR-C [5,6,25,27,28,36] or a different isoform that is initiated from the AUG codon at amino acid 301 of the full length protein sequence [7], whereas the placental cytotrophoblast is devoid of PR and the placental syncytiotrophoblast contains only this cytoplasmic isoform. By contrast, the maternal decidua contains not only PR-A, as its major isoform, but also PR-B, PR-S and the cytoplasmic isoform. These data differ from those recently reported [25] where PR-B and PR-A isoforms were observed in amnion nuclear extracts yet, paradoxically, the antibody used failed to show immunoreactive PR by immunohistochemistry and theoretically, should not detect the PR-C, PR-S, or PR-M isoforms in western blotting, as the epitope for this antibody is found only at the N-terminal region of the PR-A isoform [37]. However, this epitope is present in the PR301 protein and thus that particular antibody could be targeting this protein. In the present study, no PR-B or PR-A isoforms were detected in the amnion or chorion and the hypothesis that progesterone receptor switching occurs within these tissues at term or in labour [23] is not supported. Although, we cannot rule out the presence of a small amount of PR-B or PR-A isoforms being present in the nuclei of amnion epithelial cells, despite a lack of expression of these isoforms at the mRNA level (Figs 3 and 4), the present study indicates that PR-B and PR-A are of little importance in relation to the 60 kDa isoform in fetal membranes. Additionally, the PR immunoreactivity in the present study that we consider represents either the C or PR301 isoform, was confined to the cytoplasm of the amniotic epithelium and the chorionic cytotrophoblast, and was the major isoform present, whereas no cytoplasmic immunoreactivity was detected in the aforementioned study [25]. These data are qualitatively similar to that obtained in the baboon [38], where PR was not observed in the amnion or chorion, but strong nuclear and weak cytoplasmic staining was observed in the decidua with the JZB39 rat anti-PR monoclonal antibody. Observations obtained with JZB39 [39], which has been shown to only detect the 60 kDa isoform in myometrium and not T47D extracts by immunoblotting, were dependent upon protein concentrations [39]. These data suggest that protein loading in immunoblotting is critical for good detection of the 60 kDa isoform. Indeed, this isoform was weakly represented on western blotting in a previous study [25], whereas our data show intense expression of the 60 kDa isoform (Fig 5) for this exact reason. Alternatively, these discrepant observations may be related to the antibodies used, the use of nuclear extracts compared to whole cell extracts or the presence of contaminating cytoplasm/tissues in the previous study [25]. Indeed, there is confusion in the literature concerning the use and specificity of available PR antibodies with some antibodies lacking specificity for particular PR isoforms [7,19], despite the evidence provided by the suppliers that the antibodies are specific. For example, the C-20 polyclonal antibody we used herein is marketed showing 4 distinct bands on western blots for PR in T47D cell extracts. Yet Salamecos & Gellersen could not demonstrate any PR-specific bands in T47D cell extracts, COS-7 cells or *in vitro *translated products with the C-20 antibody [7]. We have demonstrated the same distinct 4 PR isoforms using our T47D extracts with this antibody, including a strong band for PR-B, yet no PR-B protein was present from proteins generated from the extra-embryonic tissue extracts, despite the presence of transcripts for PR-B (Figs. 3 and 4). This could be due to either the loss of translation from the AUG codon at amino acid 1 in these cells, that T47D cells differ from laboratory to laboratory or protein degradation occurred during the preparation of samples. The latter option is unlikely, because myometrial tissue prepared in the same way as the extra embryonic tissues also produced PR-B protein on our blots (Fig 5.). The conclusion, therefore, is that the 60 kDa protein detected is generated from a primary translation site in these tissues that arises from either the AUG codon at amino acids 301 or 595.

In agreement with our studies, Goldman et al. [25] showed that PR-A is expressed in the nucleus of the decidua but absent in the amnion by immunohistochemistry, but paradoxically, found all isoforms present by western blotting. By contrast, our western blot studies indicate the major PR isoform in the fetal membranes (amnion and chorion) is a 60 kDa protein that could be the PR-C isoform and that it is localised within the cytoplasm, without immunoreactivity within the nuclei of the same cells and, therefore, we consider that the relevance of the changes in levels of the PR isoforms detected within the amniotic nuclear extracts of labour [25], should be considered with caution.

Using antibodies that detect all known PR isoforms, we demonstrated that the major isoform in the amnion, chorion and syncytiotrophoblast is the 60 kDa isoform (Fig 5). This isoform was previously reported as the PR-C isoform in human breast cancer cells [5,6,28], human fetal membranes [25], the rat decidua basalis [40] and the guinea pig cervix [27], suggesting that the ~60 kDa PR protein in the amniochorion and placenta is PR-C (Fig 5). The absence of PR-B and PR-A transcripts in amniochorion, which was devoid of decidua (Figs 3 and 4), confirmed these findings. By contrast, the major isoform in the decidua is considered to be PR-A [41], but in this study PR-B, PR-S and the 60 kDa isoforms are also expressed (Figs 3, 4 and 5). In light of the study by Goldman et al., these data need further clarification, but suggest that the 60 kDa PR isoform is an important molecule in the fetal membranes, decidua and syncytiotrophoblast. These data also suggest that a re-evaluation of the roles of PR in the fetal-maternal interface is needed and the idea that PR-A simply acts as a dominant negative regulator of PR-B action is not applicable to these particular tissues [23].

The exact mechanism involved in the synthesis of the N-truncated PR isoforms that include PR-C is unknown, but limited evidence suggests that PR-C may be produced using a third oestrogen dependent promoter that replaces both exon 1 and exon 2 of the full length PGR gene [42], with an AUG codon at amino acid 595 being the translational start site in a number of mammalian and non-mammalian species [5,27,28,42-44]. The demonstration that PR-C isoforms in the human breast cancer cell do not arise from proteolysis of larger PR-isoforms [13] but from two specific PR transcripts that originate from a 11.4 kb complex [5] and that specific PR mRNA transcripts are present in human fetal membranes and placenta that are not any of the other recognised PR isoforms (this study), strongly suggests that the 60 kDa isoform is generated through promoter specific transcription in extra-embryonic tissues. The function of N-truncated PR isoforms including PR-C is unclear, although they may act as modulators of PR-A and PR-B transcriptional activities in those cells that produce these isoforms [13]. In the human amnion and chorion, dominant negative regulation of PR-B and PR-A transcriptional control seems unlikely in the absence of any measurable PR-A or PR-B isoforms and point towards PR-C specific functions. Because PR-C lacks a full DNA binding domain [13], but has a nuclear localisation signal and two dimerisation domains [42], the intriguing possibility that PR-C may associate with other transcriptional elements to modulate gene transcription is raised [13]. Indeed, evidence exists in the rat myometrium that during labour the 60 kDa PR-C isoform translocates from the cytoplasm into the nucleus [18,36]. Additionally, studies on the promoter activities of PR-B, PR-A and a truncated PR that is equivalent to PR-C have demonstrated that a PR-C-like construct has 3-times the activation potential of PR-B and 1.5-fold the activation of PR-A on stimulation with medroxyprogesterone acetate in early secretory phase human stromal cells [45] and enhances PR-B and PR-A transactivation in breast cancer cells [13] suggesting that PR-C may have gene modulatory functions that differ to those of PR-B and PR-A in certain cell types, as has been shown for the main PR isoforms in a tissue-specific context [46-48]. The 60 kDa protein identified in this study may not be the PR-C isoform that originates from the AUG codon at 595, as presented in the literature, but an isoform that originates at the AUG codon at 301 [7]. However, the weight of evidence in the literature currently suggests that it is most likely PR-C; indeed, the demonstration that protein produced from the original PR-C plasmid cloned from a cDNA library produced a 60 kDa band on western blot (Fig 4) supports this. PR-C plasmids generated from PCR products generated from a PR-B containing plasmid produce proteins of only 38 kDa [7], consistent with the molecular mass of PR-M, although the authors suggest that proteins for PR-M, PR-S, PR-C and PR-T isoforms do not exist, suggesting that more research into the nature of proteins produced from these expression plasmids is required. However, the cytoplasmic location and relatively high levels of this 60 kDa protein in the amniochorion are suggestive of a localised function in the cytoplasm rather than a nuclear genomic function for this tissue and that this localisation is important for sustained gestation. It now seems imperative that the identity, regulation and role of the 60 kDa cytoplasmic PR isoform are investigated to further our understanding of the role of progesterone in these tissues in relation to human parturition.

## Competing interests

The authors declare that they have no competing interests.

## Authors' contributions

AHT conceived the study, carried out the immunohistochemistry, RT-PCR and western blotting studies, participated in the data analysis and drafted the manuscript. PCM obtained consent, collected the clinical samples and helped to draft the manuscript. DJT participated in the design of the study and helped to draft the manuscript. SCB participated in the study design and data analysis and helped to draft the manuscript. All authors read and approved the final manuscript.
